# The Tumor Microenvironment as a Regulator of Endocrine Resistance in Breast Cancer

**DOI:** 10.3389/fendo.2019.00547

**Published:** 2019-08-08

**Authors:** María Inés Diaz Bessone, María José Gattas, Tomás Laporte, Max Tanaka, Marina Simian

**Affiliations:** ^1^Laboratory of NanoBiology, Instituto de Nanosistemas, Universidad Nacional de San Martín, Buenos Aires, Argentina; ^2^Amsterdam UMC, VUmc School of Medical Sciences, University of Vrije, Amsterdam, Netherlands

**Keywords:** breast cancer, endocrine resistance, microenvironment, immune system, extracellular matrix, carcinoma associated fibroblasts, estrogen receptor

## Abstract

Estrogen receptor positive breast neoplasias represent over 70% of diagnosed breast cancers. Depending on the stage at which the tumor is detected, HER2 status and genomic risk, endocrine therapy is combined with either radio, chemo and/or targeted therapy. A growing amount of evidence supports the notion that components of the tumor microenvironment play specific roles in response to treatment and that strategies targeting these key interactions with tumor cells could pave the way to a new generation of therapies. In this review, we analyze the evidence suggesting different components of the tumor microenvironment play a role in hormone receptor positive breast cancer progression. In particular we focus on the immune system, carcinoma associated fibroblasts and the extracellular matrix. Further insight into the cross talk between these constituents of the microenvironment and the tumor cells may lead to therapies that eliminate disseminated metastatic cells early on, and thus reduce distant disease relapse which is the leading cause of death for patients who are diagnosed with this illness.

## Introduction

Breast cancer is the most frequent cancer in women in the western world; one in eight women will have breast cancer at some point in their life (1). Seventy-five percent of diagnosed breast tumors express estrogen receptor-alpha (ERα) and endocrine therapy is the treatment of choice for patients with tumors of these characteristics. Within the scope of endocrine therapies, tamoxifen, a selective estrogen receptor modulator (SERM), has been the most widely used over the last 30 years (2). However, today other options are available such as aromatase inhibitors (AI) and selective estrogen receptor downregulators (SERDs) such as Fulvestrant (3). Standard-of-care regimens have not been established for ER+ tumors. Depending on the stage at which the tumor is detected, HER2 status and genomic risk, endocrine therapy is combined with either radio, chemo and/or targeted therapy (4). Patients with ER+ early stage breast cancer are susceptible to late recurrence that can take place even after 15 years of treatment interruption. Several strategies have been studied to prolong adjuvant endocrine therapy from 5 to 10 years, including 10 years of therapy with tamoxifen, 10 years of tamoxifen with an AI, or a 5-year period of tamoxifen followed by 2–5 years of treatment with an AI. Overall, the best outcomes with extended therapy in patients with a high risk of relapse are found in those who have received 5 years of tamoxifen and up to 3 years of an AI (5, 6).

## Hormones and ER-α

**Figure 1:**
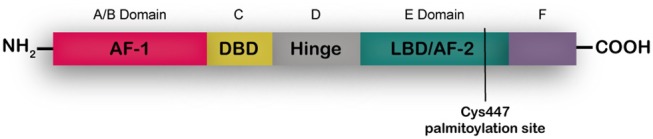
ERα structume. The functional domains ERα include the DNA binding domain (DBD), ligand binding domain (LBD), and two transcriptional activation functions (AF), the AF-1 ligand-independent activation function domain and the AF-2 ligand-dependent activation domain. The A/B domain, at the amino terminus of the protein, contains AF-1. The C domain binds to DNA motifs (EREs) at target genes. The D domain or hinge region contributes to DNA binding specificity and nuclear localization. The E domain or LBD interacts with estrogens or SERMs. At the C- terminus is the F domain.

ERα is a transcription factor present in different adult tissues such as mammary gland, ovaries, uterus and brain (7, 8) regulating cell proliferation, migration and survival. In the breast ERα controls development and plays a key role in tumor growth as mentioned above (9). Full length ERα is a 66-kDa ligand-dependent transcription factor that is activated by 17-β-estradiol. ERα shares a common structural organization with other steroid hormone receptors consisting of two transcriptional activation domains, the AF-1 N-terminal ligand-independent activation function domain and the AF-2 C-terminal ligand-dependent domain. A ligand-binding domain (LBD) also resides in the C-terminal region, and the DNA-binding and hinge domains are located in the central region of the protein (Figure 1). A palmitoylation site is found at Cys447 within the AF-2 domain (8, 10). In breast cancer cells two shorter isoforms of ERα have been described, ERα46 that lacks the A/B domain (11) and ERα36 that lacks both AF-1 and AF-2 transactivation domains and has an extra 27aa c-terminal domain (12, 13). According to the accepted mechanism of action, unbound full length ERs are located as monomers mostly in the nucleus and dimerize upon steroid binging. A percentage of the receptor sits in the cytoplasm bound to HSP90, that is released upon estrogen binding, enabling dimerization and traslocation of the receptor to the nucleus. In the nucleus, dimerized ERs bind estrogen-response-elements in the promoter regions of target genes regulating their transcription. ERα can also act as a co-regulator of other transcription factors such as AP-1, SP-1, and NF-κB especially when increased tyrosine kinase activity leads to phosphorylation of the AF-1 N-terminal region. Palmitoylated ERα localizes at diverse extranuclear compartments, including the plasma membrane, and is proposed to mediate rapid, non-genomic actions of ERα in the context of a cross talk with tyrosine kinase membrane receptors such as EGFR (14). It is now well-accepted that it is the same nuclear ERα that is modified and localizes at the membrane. In depth reviews of ER signaling have been recently published elsewhere (10, 15).

Endocrine therapy for the treatment of breast cancer, in the form of tamoxifen, was the first targeted therapy to be developed (2). Tamoxifen is classified as a SERM because it modulates ER's activity by leading to the recruitment of co-inhibitors when the receptor binds 17-β-estradiol and thus impairs its transcriptional activity. Aromatase inhibitors inhibit the local synthesis of estrogens and are recommended for postmenopausal patients (16). SERD's, like Fulvestrant, lead to the degradation and dowregulation of the bound receptor (17).

While endocrine therapy is the most effective treatment for ER+ breast cancer, its effectiveness is limited by considerable rates of *de novo* (intrinsic) and acquired resistance. It is estimated that 50% of patients with metastatic disease will not respond to endocrine therapy and 30% of patients with early disease will eventually relapse having initially responded to therapy (18). Thus, understanding what leads to resistance is of great clinical importance considering that about 12% women in the U.S. are expected to have breast cancer at some point in their life (1).

Distant disease relapse is the main cause of death for patients who are diagnosed with early stage breast cancer. Breast cancer cells spread and settle in other tissues in small foci called micrometastasis. Evidence suggests that cells leave the primary tumor in the initial stages of tumor development (19). Genomic profiling of disseminated tumor cells in bone marrow shows that they are less genomically aberrant and have fewer copy number alterations than their corresponding primary tumors (20). Cells within micrometastasis can remain dormant for many years (21). The mechanisms involved in maintaining the dormant state and what actually triggers the onset of proliferation and the development of overt metastasis is still under debate. Interestingly, even though ER+ breast tumors are a perfect example of this clinical scenario, very few papers have actually addressed the mechanisms that are involved in recurrence in the context of endocrine therapy. Clinical data reveal that more than half of the recurrences of ER+ tumors take place 5 years or longer after diagnosis and surgery of the primary tumor; some patients experience recurrence after more than 20 years (22). The fact that prolonging adjuvant treatment from 5 to 10 years reduces recurrence and death between years 10 and 15 strongly suggests that blocking ER signaling maintains cell proliferation suppressed and impedes the exit from the dormant state. In this sense, Ogba et al. recently showed that breast cancer metastases and tumor arousal from dormancy are promoted by direct actions of estradiol and progesterone on the malignant cells (23). Through a series of elegant experiments using ovariectomized nude mice and four breast cancer cell lines that differed in their levels of expression of ER, PR, and CK5 they showed that ER+ PR+ luminal tumor cells can seed distant organs, where they remain dormant as micrometastases and sheltered from therapies but arousable by hormone repletion. Interestingly, as demonstrated in human clinical samples, the micromestastasis were composed of heterogeneous cell populations even though pure luminal cells were initially inoculated into the mice, to the best of the researchers' knowledge (23). In another study, the expression of HER2, ER, PR, Ki-67, and CK5 were studied in 72 primary breast cancers and their corresponding metastatic lesions (24). In accordance to previous studies, ER expression in the primary tumor was associated to late recurrence when the tissue was also positive for Ki67 (24). Hess and collaborators showed, when analyzing the distinct patterns of relapse of 558 patients, that rates of recurrence were significantly higher in patients with ER-negative status for the first 2 years of follow-up, but not thereafter (25). ER+ tumors showed increased recurrence in bone whereas ER- cancers were found to metastasize preferentially to viscera and soft tissues (25).

Even though loss of ER expression may account for endocrine resistance, this phenomenon is observed in 15–20% of patients with progressed metastatic disease (26). Mutations in ERα are rare in primary tumors; only 0.5% of luminal breast cancers reveal mutations in ERα, and amplifications are observed in 2.6% of cases (27). In the metastatic setting however, a higher frequency of ERα mutations are observed, representing approximately 20% of cases (28, 29). Most mutations are found in the ligand binding domain of the receptor and are associated with the agonist conformation of the receptor as determined by biochemical and structural studies (29). The fact that mutations are found in progressed tumors suggests that they are the result of the expansion of rare clones in response to the selective pressure generated from targeted therapies against ER signaling (30).

Thus, evidence strongly supports the notion that ER signaling is critical even in later stages of tumor progression, years after initial diagnosis, surgery and prolonged treatment with endocrine therapies.

## The Microenvironment as a Key Player in Endocrine Resistance

A growing body of evidence supports the notion that components of the tumor microenvironment play specific roles in response to therapy and that strategies targeting the interactions established with tumor cells could pave the way to a new generation of therapies. Carcinoma associated fibroblasts (CAFs), adipocytes, immune cells, endothelial cells, pericytes, the extracellular matrix (ECM) and soluble factors all contribute to tumor evolution (31–36). Thus, the resulting progression of a tumor is a consequence of the sum of interactions that are generated by all these players together with the tumor cell population. In ER+ breast cancer in particular, we are only now starting to understand the role played by the tumor stroma in response to therapy. The studies that will be reviewed in the following sections are, to our knowledge, those that have tackled how diverse microenvironmental players impact on the biology of ER+ tumors. However, it is important to understand that this is probably only the tip of the iceberg and that far more sophisticated interactions between these stromal players underlie the response to therapy.

## The Immune System

Inflammation, one of the hallmarks of cancer, is associated to breast cancer development and progression. Studies show that regular use of non-steroidal anti-inflammatory drugs (NSAIDS), such as aspirin, significantly decrease the risk of ER-positive but not ER-negative breast cancers (37). Involution after pregnancy and obesity are two examples of risk factors associated to an inflammatory microenvironment and progression of breast cancer. In the case of pregnancy, involution, which follows pregnancy and takes place when lactation ends is considered to be responsible for the increase in breast cancer risk that has been described in the 10-years that follow parturition (38). The increased risk and poor prognosis of pregnancy associated breast cancer is thought to be associated to the inflammatory mediators that are present and active during involution. Extensive immune infiltration is present during involution, similar to what is observed during wound-healing (38). The ER status of pregnancy associated breast cancer is not clear (39). Although some studies suggest that there may be a reduced number of ER+ breast tumors amongst this population, others suggest that the high local and circulating levels of estrogens are responsible for the downregulation of ER and PR (39).

In postmenopausal women weight gain is associated to an increased risk of ER+ breast cancer (40, 41). Moreover, obese women with ER+ breast cancer have a higher rate of recurrence than lean women after treatment with endocrine therapy (42) and obesity on its own is related to tamoxifen resistance (43). Recruitment of macrophages into adipose tissue is a characteristic of obesity induced inflammation. Adipocytes and macrophages interact and have been shown to lead to activation of the proinflammatory transcription factor NF-κB. The degree of infiltration of macrophages is associated to the development of tamoxifen resistance (43).

Several studies suggest that tumor associated macrophages protect cancer cells from the anti-tumor immune responses. Macrophages isolated from mouse and human tumors can directly suppress T cell responses *in vitro* (44), and depletion of macrophages enhance CD8+ T-cells in a model of breast cancer under chemotherapy (45). A recent study analyzing circulating M2-like monocytes in breast cancer patients showed that they were increased in this population in comparison to healthy controls and patients with benign lesions (46). Another study evaluated the relationship between CD204 expression on tumor associated macrophages and clinicopathological factors in patients with invasive breast cancer. The authors found that in a sample of 108 luminal-like tumors high expression levels of CD204 was associated to decreased relapse-free survival and distant relapse-free survival (47).

Cytotoxic T cells, recognizable by the expression of CD8, play a major effector role in the adaptive immune system. Cells that present foreign antigens in association with the major histocompatibility complex class I molecule are recognized by cytotoxic T lymphocytes through a specific interaction between the T-cell receptor and the presented antigen. This interaction causes the activated T cell to release proteins such as perforin and granzyme that lead to cell death through lysis of the cell membrane [23]. These mechanisms can act on malignant cells which, unlike their normal counterparts, present atypical antigens [24, 25]. A recent paper analyzing 12,439 tumor samples, 8,775 of which were ER+ showed that for ER+ tumors that express HER-2, the presence of intratumor CD8+ T cells was associated with a 27% reduction in the hazard of dying from breast cancer (48). The analysis of the expression levels PD-L1 revealed that in the case of ER+ tumors, 20% of patients show detectable levels (49) compared to around 58% in the case of triple negative breast cancer (50). Response to anti PD-1/PD-L1 monotherapy in metastatic breast cancer showed durable clinical benefit for patients with triple negative breast cancer (50, 51). Regarding patients with ER+ tumors, few studies have specifically addressed this tumor type. Recently, Rugo et al. analyzed the antitumor activity of Pembrolizumab, an anti-PD-1 monoclonal antibody, in patients with ER+/Human Epidermal Growth Factor Receptor 2–negative advanced breast cancer (49). The objective response rate was 12% and a durable clinical benefit of more than 24 weeks was observed in 20% of patients (49). Another recent study was carried out analyzing 61 primary breast cancer tissues, 85% of which were ER+. Only eight of the samples were triple negative and one was ER-/HER2+ (52). The authors characterized the CD8+ tumor infiltrating lymphocytes and found that they retained robust capacity for production of effector cytokines and degranulation capacity even though they expressed PD-1, a hallmark of exhaustion (52). Additionally, they showed that CD8+ tumor infiltrating lymphocytes treated with CD3:CD19 bi-specific antibodies were able to kill breast cancer cells as efficiently as peripheral blood mononuclear cells from the same patients (52). The retention of polyfunctionality therefore implies the possibility that they are mostly composed of bystander T cells. This may explain the lack of impressive clinical responses to checkpoint blockade therapies in breast cancer.

**Figure 2:**
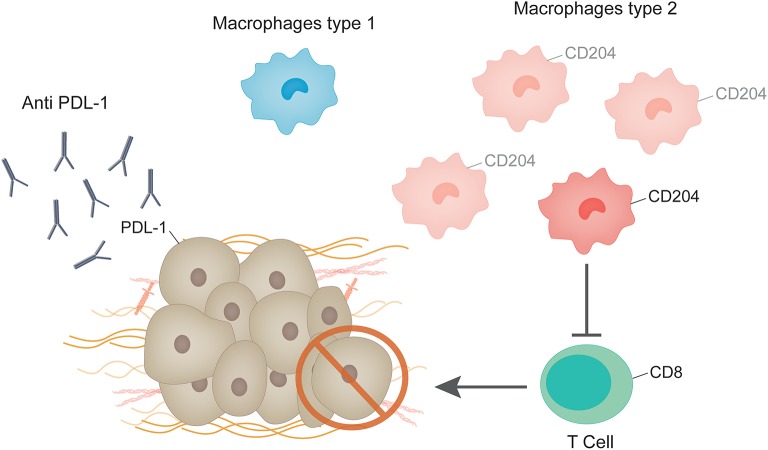
Immune system and ER+ breast tumors. Tumor associated macrophages protect cancer cells from the anti-tumor immune responses. M2 macrophages are increased in tumors in comparison to healthy controls and patients with benign lesions. CD204 expression in tumor associated macrophages is enhanced in patients with invasive breast cancer. Macrophages isolated from mouse and human tumors can directly suppress T cell responses *in vitro*, which are recognizable by the expression of CD8. Response to anti PD-1/PD-L1 monotherapy in metastatic ER+ breast cancer showed durable clinical benefit in a low percentage of patients, although very few studies have addressed this issue.

In studies using breast cancer cell lines, ERα was shown to be a negative regulator of PD-L1 gene transcription as revealed by the mutually exclusive expression pattern of ERα and PD-L1 (53). Moreover, analysis of TCGA data derived from human breast cancer samples demonstrated that the average PD-L1 mRNA levels of ERα-positive tumors were significantly lower than those of ERα-negative tumors (53). Thus, immune response and ER signaling do not seem to be completely independent phenomenon. In this line of thought, one paper shows that antiestrogens induce immunosuppression in the tumor microenvironment, through a TGFβ-dependent mechanism contributing to the development of antiestrogen resistance in breast cancer (54). The impact of tamoxifen on the immune system has been reviewed in Behjati and Frank (55). Further studies analyzing the impact of anti-estrogen treatments on the interaction of CD8+ tumor infiltrating lymphocytes, macrophages and ER+ breast cancer cells may shed light on the development of future therapeutic strategies for breast cancer patients (Figure 2).

## Carcinoma Associated Fibroblasts

In the normal mammary gland, fibroblasts in the stroma are located in close proximity to the epithelial ducts. They play a key role during puberty producing soluble factors, ECM components and proteases that are involved, amongst other things, as intermediaries in hormone signaling (56). Response to estrogens during development is the result of an intricate cross-talk established between ER+ sensor epithelial cells and stromal fibroblasts (56, 57). During ductal elongation, estrogens induce expression of amphiregulin that acts on EGFR positive stromal cells. In response, fibroblasts produce factors such as fibroblast growth factors and insulin-like growth factor-1 that signal back to the epithelium inducing proliferation (56). In ER+ breast cancer, in contrast to what is proposed for the normal mammary gland, estrogens impact directly on the proliferation of the ER+ tumor cells (15). Together with this, tumor progression is associated to changes in the stroma that contribute in the promotion of the malignant phenotype by secretion of additional growth stimulatory, angiogenic, immune-regulatory and pro-invasive soluble factors (58). A convincing link between tumor progression and the underlying stroma was elegantly demonstrated by Park's group where differential gene expression from the tumor stroma was shown to generate clusters linked to clinical outcome in breast cancer, independently of breast cancer subtypes (59). In the context of response to endocrine therapy we previously showed that soluble factors found in conditioned media derived from CAFs induce tamoxifen resistance in a murine model of ER+ breast cancer (60). Growth factors, proteases and signaling through β1 integrin were found to be involved in the protective effect the CAFs induced over the malignant cells exposed to endocrine therapy (60). In another study, using primary human CAFs co-cultured with MCF-7 cells, two CAF populations were identified through differential expression of CD146 in human breast tumors (61). CD146 (MCAM) is a stromal surface marker that defines fibroblast subtypes in the hematopoietic stem cell niche (62). These subtypes differentially influence the fate of peripheral blood monocytes (62). In the context of breast cancer, CD146^−^ CAFs were shown to inhibit ER expression in MCF-7 cells, reduce sensitivity to estrogen, and increase resistance to tamoxifen. On the other hand, the presence of CD146^+^ CAFs stimulated ER expression and sustained estrogen-dependent proliferation and sensitivity to tamoxifen. Conditioned media from CD146^+^ CAFs reestablished tamoxifen sensitivity to tamoxifen-resistant breast cancer cells. Gene expression profiles of patient breast tumors with predominantly CD146^−^ CAFs correlated with decreased clinical response to tamoxifen and worse patient outcomes (61). In the same line of thought, using a novel microfluidics-based organotypic model, Morgan et al. showed that when MCF-7 cells are co-cultured in 3-dimensions with immortalized human mammary fibroblasts, ER transactivation was increased in the presence of 17-β-estradiol. Moreover, the incorporation of fibroblasts increased the speed of development and size of estrogen-induced hyperplasias. This phenomenon was associated to reduced apoptosis in the co-culture model (63). Other publications have shown regulation of ER signaling in MCF-7 and T47D cells using either immortalized skin fibroblasts or marrow-derived stromal cells (64, 65). In first case the authors show that CAFs induce tamoxifen resistance by increasing mitochondrial activity in breast cancer cells. In the second case, paracrine stromal signaling leads ER downregulation in MCF-7 and T47D cells.

Another recently unraveled mechanisms of therapy resistance involves exosome transfer from stromal to breast cancer cells (66). In the context of ER positive breast cancer, transfer of OncomiR-221 containing microvesicles from CAFs to breast cancer cells has been shown to induce the expansion of cancer stem cells with increased self-renewing capacity, and resistance to endocrine therapy. Interestingly, tamoxifen has been previously shown to lead to the enrichment of breast cancer stem cells both in human and murine models, as well as in primary patient tissues (67–70).

**Figure 3:**
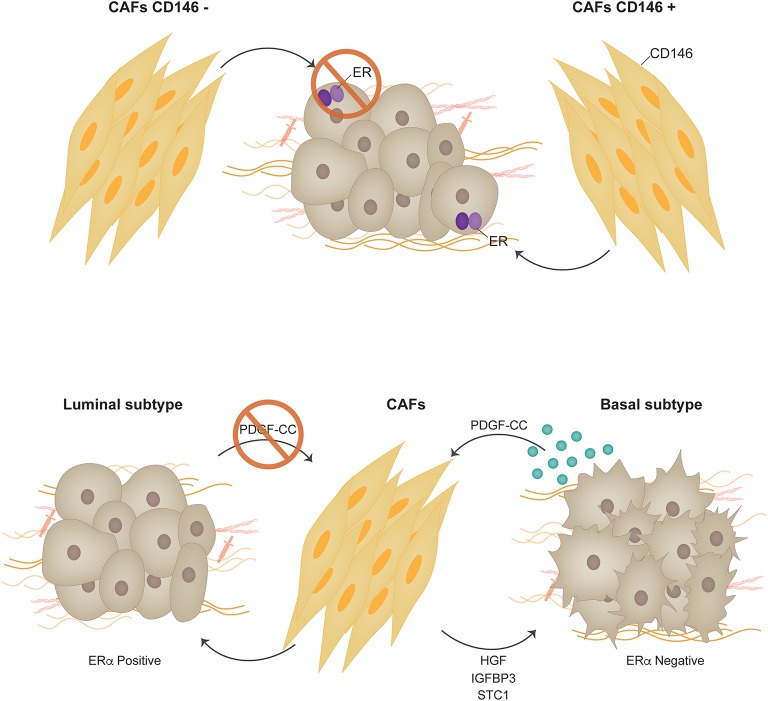
Carcinoma associated fibroblasts. Two CAF populations were identified through differential expression of CD146 in human breast tumors. CD146 is a stromal surface marker that defines fibroblast subtypes in the hematopoietic stem cell niche. In the context of breast cancer, CD146^−^ CAFs were shown to suppress ERα expression in MCF-7 cells, decreased sensitivity to estrogen, and increase resistance to tamoxifen. On the other hand, the presence of CD146^+^ CAFs promoted ERα expression and sustained estrogen-dependent proliferation and sensitivity to tamoxifen. PDGF receptors and ligands have recently been shown to actually modulate whether a tumor is luminal or basal and thus whether it may or not respond to endocrine therapy [Permission obtained from Springer Nature (73)].

Recent studies question the distinct origin of luminal and basal-like breast tumors and suggest a high degree of plasticity and heterogeneity between these two tumor types (71). Interconversion of luminal or basal-like tumor cells has been demonstrated to occur efficiently *in vitro*, establishing a common progenitor cell origin (72). PDGF receptors and ligands have recently been shown to actually modulate whether a tumor is luminal or basal and thus whether it may or not respond to endocrine therapy. Roswall et al. showed that CAFs act as determinants of the molecular subtype of breast cancer (73). Previous studies showed an association between PDGF-CC ligand expression in breast tumor cells and the triple negative subtype (74). Further insight revealed that PDGF-CC is an independent prognostic factor for poor survival in breast cancer (73). Experiments using triple-negative patient-derived xenografts (PDX), MDA-MB-231 triple negative breast cancer cells orthotopically inoculated in immunocompromised mice and FVB/N mice bearing orthotopically transplanted tumors from MMTV-PyMT; Pdgfc^+/+^ or MMTV-PyMT; Pdgfc^−/−^ mice were shown to upregulate ERα expression when PDGF-CC was downregulated or inhibited and consequently respond to tamoxifen. PDGFRα and PDGFRβ were found to be expressed in the CAF compartment in all analyzed tumors (74) strongly indicating that the paracrine mode of signaling by PDGF-CC is from the epithelium to the stroma, and not autocrine within the stromal compartment. As a consequence of PDGF-CC signaling CAFs have been shown to produce molecules such as HGF, IGFBP3 and STC1 that are postulated as candidate mediators of the induction of the luminal phenotype (73) (Figure 3).

CAFs could be thus thought of as putative therapeutic targets to indirectly modulate the tumor epithelial compartment. Further insight in the understanding of the mechanisms implied in stromal epithelial interactions may produce a paradigm shift in the way we think about breast cancer classification and treatment.

## The Extracellular Matrix

A central component of tumor tissue is the ECM that has in the last years been recognized as a key player in tumor progression and resistance to therapy in various malignancies, including breast cancer. It is well-accepted that tumors behave like wounds in the sense that the tumor host microenvironment is constantly in a fibrotic repair state. Changes in stromal composition and rigidity accompany breast cancer progression (75). In the normal mammary gland, the basement membrane clearly separates the epithelial compartment from the stroma. Laminin, collagen IV, fibronectin and entactin are the mayor constituents of the basement membrane that is produced jointly by epithelial, endothelial, and stromal cells. The interstitial ECM is composed of fibrillar collagens, fibronectin, glycoproteins and proteoglycans (75). The spacial arrangement together with the physical properties of the ECM determine tissue architecture and integrity. The biochemical characteristics of the matrix provide cues that modulate how the cells respond to different soluble factors such as hormones, polypeptide growth factors and chemokines.

The normal mammary gland is a soft and compliant tissue that upon malignant progression stiffens (76). This stiffening is accompanied by changes in the biochemical properties of the matrix. Remodeling of the tumor ECM involves continuous synthesis of matrix proteins, their assembly and crosslinking, as well as their turnover by proteases. This remodeling contributes to ECM stiffening, which is the consequence of increased collagen deposition, enhanced collagen crosslinking (as a result of lysyl oxidase (LOX) enzyme expression), and the reorientation of the collagen fibers to a parallel disposition (77–79). Significantly, increased collagen abundance and reorganization into thick, linearly oriented fibers correlates with tumor progression and clinical outcome (80). High ECM stiffness may also predispose individuals to develop certain types of cancer. Normal breast tissue clinically determined to have high mammographic density contains stiffer ECM, thicker collagen fibers and more linearized collagen than low mammographic-dense breast tissue (80), and was shown to increase the overall lifetime risk of breast cancer development (81). Tissue stiffness has been associated to breast cancer progression (82). Moreover, it is directly associated to response to chemotherapy (83, 84). Interestingly, reports analyzing tumor progression and stiffness suggest that tumors that have nodal metastasis usually have a stiffness >150 kpa (85).

An association between breast cancer progression and matrix composition analyzing human tumor samples was suggested as early as 2002 (86). However, the work of Els Burns's group was the first, to our knowledge, to focus on ER+ tumors. They showed a convincing association between an ECM gene cluster and disease progression in ER+ tumors derived from patients treated with tamoxifen (87). The authors examined 112 ER-positive primary breast carcinomas from patients with advanced disease and clearly defined therapy response types (i.e., 52 patients with objective response vs. 60 patients with progressive disease) from start of first-line treatment with tamoxifen. Eighty-one genes were found to be differentially expressed between the tamoxifen sensitive and the resistant tumors. From the 81 genes, 44 were extracted and validated on an independent set of 66 tumors. Within the group of identified genes, a cluster of ECM genes was identified: TIMP3, FN1 (fibronectin 1), LOX, COL1A1 (collagen type 1 alpha 1 chain), SPARC, and TNC (tenascin C). In all cases the overexpression of the genes was associated to disease progression. A second study by the same research group focused on these 6 genes and investigated in a sample of 1,286 tumors whether the mRNA expression levels were associated with the evolution of the disease, i.e., prognosis (independent of therapy response), clinical benefit from therapy with tamoxifen, or both (88). The results showed that high expression levels of FN1, LOX, and SPARC were associated with shorter metastasis free survival in lymph node negative patients who received no adjuvant systemic therapy. Other studies have analyzed the expression levels of FN in formalin fixed sections and also suggest that FN is associated to disease progression in breast cancer (86, 89–92).

**Figure 4:**
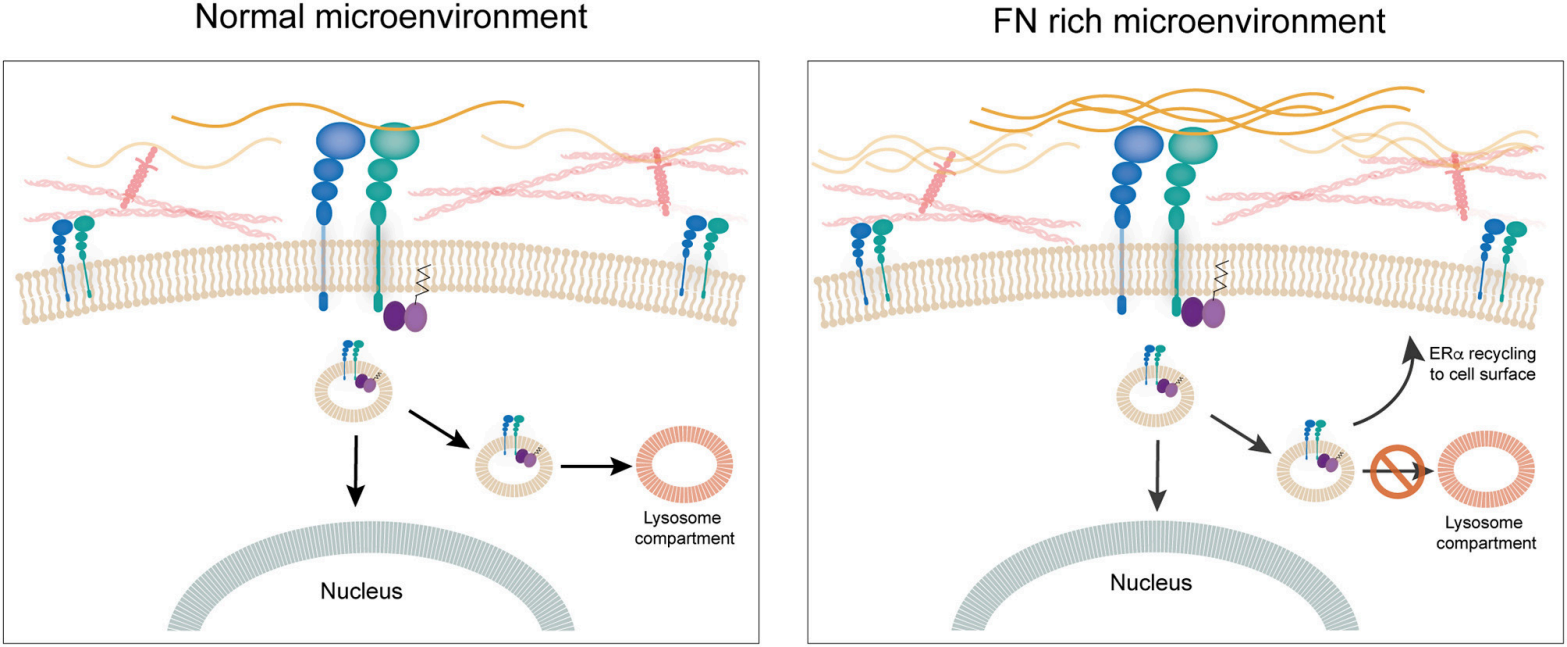
The extracellular matrix. FN is associated to disease progression in breast cancer. Culturing ERα+ human breast cancer cells on FN leads to endocrine resistance through binding to β1 integrin. When cells are in contact with FN, ERα is not downregulated after 1 h treatment with estradiol. Estradiol induces endocytosis in breast cancer cells and ERα located at the cell membrane travels in endosomes to the nucleus. When FN is present, ERα is endocytosed and “dragged” by β1 integrin back to the cell surface, apart from going to the nucleus. Also, there is no co-localization of ERα with the lysosomal compartment strongly supporting the notion that the β1 integrin/FN interaction directs the fate of ERα and thus response to tamoxifen.

Our research group has been working on establishing a mechanistic link between ECM components and response to tamoxifen in ER+ breast cancer cells. We previously showed that culturing ER+ human and murine breast cancer cells on FN leads to endocrine resistance through binding to β1 integrin (60). We also showed that FN induces phosphorylation of ERα in serine-118, a site that has been associated previously to ligand-independent activation of ER transcriptional activity and tamoxifen resistance (93, 94). To unravel the mechanism behind the induction of tamoxifen resistance by FN we looked into the dynamics of ERα recycling for cells cultured on FN as compared to bovine serum albumin. Surprisingly, we found that when cells are in contact with FN, ERα is not downregulated after 1 h treatment with estradiol. Further studies led us to find that estradiol actually induces endocytosis in breast cancer cells and that it is ERα located at the cell membrane that travels in endosomes to the nucleus (95). Previously, others had shown an association of ERα to the endosomal compartment (96), but induction of endocytosis by estrogens had been only described for neurons (97), to our knowledge. Inhibition of endocytosis by both pharmacological and genetic approaches led to inhibition of ER's transcriptional activity. Importantly, what our work shows is that when FN is present, ERα is endocytosed and “dragged” by β1 integrin back to the cell surface, apart from traveling to the nucleus (95). Moreover, in these conditions there is no co-localization of ERα with the lysosomal compartment strongly supporting the notion that the β1 integrin/FN interaction directs the fate of ERα and thus the response to tamoxifen (95). The fact that membrane ERα is critical for the transcriptional activity in breast cancer cells had already been strongly suggested by previous work of Filipo Acconcia and collaborators (96, 98). Our work confirms their findings and unravels a key role for an ECM component in the regulation of ERα's half-life and transcriptional activity (95). Thus, the ECM appears to have a central role not only through the establishment of mechanical cues, but by directly regulating the impact of hormone-action in breast cancer cells. Further studies are needed to understand whether integrins such as β1 interact with other hormone receptor and impact on their fate and signaling capacity (Figure 4).

## Conclusion

The current management of ER+ breast cancer is based on how far the disease has progressed at the time of diagnosis, HER2 status and genomic risk leading to treatments that include chemotherapy, endocrine therapy, and targeted therapy (6). Women who are diagnosed with early stage breast cancer and can access adequate therapy have a 90% chance of being cured and multiple treatment options (6). However, for those who are diagnosed later the scenario is not that easy to manage. Targeted therapies are centered on the tumor cells. However, there is growing evidence that the tumor microenvironment plays a pivotal role in tumor progression and response to therapy. Immunotherapy is in its early years and is proving to revolutionize cancer treatment in general and impacting breast cancer management in particular. We propose that a deeper understanding of the role played by different components of the tumor microenvironment, especially focused on the niche where micrometastasis sit, may lead to the development of new therapies that could eliminate these residual cells early during treatment and thus reduce the late recurrences that characterize ER+ breast cancer.

## Author Contributions

All authors listed have made a substantial, direct and intellectual contribution to the work, and approved it for publication.

### Conflict of Interest Statement

The authors declare that the research was conducted in the absence of any commercial or financial relationships that could be construed as a potential conflict of interest.
